# Static Analysis of Information Systems for IoT Cyber Security: A Survey of Machine Learning Approaches

**DOI:** 10.3390/s22041335

**Published:** 2022-02-10

**Authors:** Igor Kotenko, Konstantin Izrailov, Mikhail Buinevich

**Affiliations:** 1 Computer Security Problems Laboratory, St. Petersburg Federal Research Center of the Russian Academy of Sciences, 199178 Saint-Petersburg, Russia; 2 Department of Secure Communication Systems, The Bonch-Bruevich Saint-Petersburg State University of Telecommunications, 193232 Saint-Petersburg, Russia; konstantin.izrailov@mail.ru; 3 Department of Applied Mathematics and Information Technologies, Saint-Petersburg University of State Fire Service of EMERCOM of Russia, 196105 Saint-Petersburg, Russia; bmv1958@yandex.ru

**Keywords:** IoT systems, cyber security, static analysis, machine learning, analytic model, survey model, formalization

## Abstract

Ensuring security for modern IoT systems requires the use of complex methods to analyze their software. One of the most in-demand methods that has repeatedly been proven to be effective is static analysis. However, the progressive complication of the connections in IoT systems, the increase in their scale, and the heterogeneity of elements requires the automation and intellectualization of manual experts’ work. A hypothesis to this end is posed that assumes the applicability of machine-learning solutions for IoT system static analysis. A scheme of this research, which is aimed at confirming the hypothesis and reflecting the ontology of the study, is given. The main contributions to the work are as follows: systematization of static analysis stages for IoT systems and decisions of machine-learning problems in the form of formalized models; review of the entire subject area publications with analysis of the results; confirmation of the machine-learning instrumentaries applicability for each static analysis stage; and the proposal of an intelligent framework concept for the static analysis of IoT systems. The novelty of the results obtained is a consideration of the entire process of static analysis (from the beginning of IoT system research to the final delivery of the results), consideration of each stage from the entirely given set of machine-learning solutions perspective, as well as formalization of the stages and solutions in the form of “Form and Content” data transformations.

## 1. Introduction

A topical problem in today’s world is the lack of IoT system (IoTS) security against the actions of intruders. IoTS security is significantly affected by software, where vulnerabilities lead to violations of confidentiality, integrity, and the availability of information [1,2]. While previously only individual files or software components were investigated to counter such breaches [3], currently a key requirement is the static (SA) and dynamic (DA) analysis of the entire information system.

IoTS consists of a great amount of data, documents and software of various types and purposes. Almost every one of these components can be a source of threats: executable code (e.g., exe files) may contain program bookmarks; Web-server code (e.g., PHP files) may contain backdoors; a hidden channel for leaking confidential and malicious information may be organized by means of stego attachments in images (e.g., jpeg files), and so on [4].

IoTS analysis is directly related to the study of its objects from different perspectives to gain new knowledge about the information security (IS) state of the system [5,6]. A great number of IoTS files, large volume of information in the files, great heterogeneity of information, container structure of documents, etc. do not allow for expert security analysis to be produced in an adequate time.

Classical automation with strict rules does not give the required results, because most of the manual work is spent on creating and debugging such rules. Therefore, an interesting and promising solution is the use of machine learning (ML), which features the transfer of human intellectual activity (clearly, resource-intensive) to artificial (software-implementable and therefore less resource-intensive) intelligence.

The application of DA is more justified for separate programs, which can be repeatedly executed in the same conditions. Full coverage of the code in the investigation process will require using all possible scenarios of the program functioning. As a result it will be possible to obtain error reports, execution logs or program results for further analysis (manually or automatically).

For large IoTS, however, this approach will be a significant problem, since the system components are in a constantly changing environment (which is extremely difficult to replace with a virtual one), and covering even part of their program code may require enormous time resources. Thus, the authors consider static analysis to be the most promising for the development of analysis applicable to modern IoTS.

Based on the above, we propose a hypothesis of the current research: ML is applicable to SA of IoTS systems for information security. This paper is devoted to its confirmation. The article proposes to limit the consideration of the SA task only, and the DA task is considered by the authors to be the direction of future research.

Despite the relative novelty of ML applications in the IS field, they have already shown relevance in this matter: for finding vulnerabilities in source [7] and machine code [8,9], attack detection in networks [10], predicting balances on components of large distributed systems [11,12], anomaly detection in real-time data [13], etc. Since a direct search for vulnerabilities is only one of IoTS analysis’ subtasks (albeit most frequently reflected in scientific publications), it is necessary to consider an application of ML on the full cycle of system investigation, not just for executable files.

### 1.1. Novelty

The novelty of the current review is as follows. First, the predominant majority of existing reviews from the software IS field are devoted to various methods of analyzing software code (source, machine, and byte code) built on the basis of ML. For example, work [8] analyzes binary code exclusively, work [7] analyzes source code, and work [9] analyzes any of these code representations. While code analysis is a central part of the search for vulnerabilities in IoTS software, the processes of data collection and preparation that precede it, as well as the subsequent visualization of the results [14], are also essential parts of a full IoTS SA.

From this point of view, the current review differs from the previous ones in that it covers the entire SA process—from the beginning of investigation of an “unfamiliar” IoTS to the moment of providing all the results of work in the form required for further processing (human or automatic). Therefore, the novelty of the current work lies in the uniform study of the application of machine learning to the entire SA process, and not only to one of its stages.

Secondly, the existing reviews mainly aim at highlighting the entire variety of ways to solve a problem (typically, code analysis) from the point of view of many ML methods and models mentioned in the articles. Thus, papers [7,8,9] mention the ML problems considered in the current review as well, with various degrees of exhaustiveness; however, no complete systematization on such problems has been made.

The current review initially defines a certain set of problems (and, accordingly, their solutions by methods from the ML field) and performs the selection and description of articles based on their applicability for SA. From this position, the current review uses the task-oriented classification of ML methods, thus, carrying out a certain systematization of the subject area objects. Consequently, in the current work, for the first time, all possibilities (both theoretical and practical) of applying the entire variety of solutions in the field of ML at each stage of SA are considered.

Thirdly, in addition to the verbal description of SA, it is not only divided into stages, but, for the first time, it is proposed to represent their actions in a formalized form—as transformations of Form and Content of the studied data in IoTS. In contrast to this approach, the paper [8] only records a formalized form of the considered specific solutions, the paper [7] gives some formulas from the ML field, and the paper [9] does not contain any formulas at all.

As a consequence, using this mathematical apparatus, a formalized presentation of detailed SA techniques for a particular IoTS (taking into account the features of its elements, having non-linear and more complex conduct schemes, as well as having the possibility of automatic verification for correctness and optimization) is possible in the future. Consequently, a new mathematical apparatus for the formalization of SA stages is proposed.

Thus, there are no works on the topic of the article (SA with ML of IoTS), and the taxonomy of close surveys is inapplicable. The taxonomy proposed in the article is aimed at systematizing all SA stages and ML tasks for IoTS. Thus, the whole set of already existing or only proposed methods is covered.

### 1.2. Contributions

The main contribution of the current review to the theory is the systematization of the basic elements of the SA (stages) and ML (task solutions) fields, in the form of two models: the SA model and the ML application model for SA. Thus, these two models cover, from different positions, the subject area delineated by the SA stages from the position of the basic tasks and ML methods. The models have a matrix form, in which the columns represent the stages and the rows define the tasks. The SA model can be used to formally evaluate the performance and efficiency (as well as other characteristics) of the algorithms used to implement a given SA step with ML solutions.

The contribution of the current review to the practice is the substantiated possibility to create an intelligent framework, which contains a whole set of elements for the construction of methods of nonlinear variations of SA for complex IoTS, while being amenable to complete automation. Thus, in the simplest case, these elements of the Framework can correspond to the cells of the specified matrix models, and with further development, have more detail. The possibility of practical application for almost any of the ML solutions in any of the SA stages is substantiated. The SA model can serve as a theoretical basis for practical implementations of the corresponding algorithms.

### 1.3. Article Content

The article has the following content. Section 3 reviews publications with independent reviews of articles on the IoTS SA process produced using ML methods. The methodological scheme of the study is constructed, reflecting also its ontology. In Section 4, the main stages of SA are defined, and their formal representation is given in the form of transformations performed on the Form and Content of the IoTS data under study. Then, typical tasks solved with ML are considered, which are also written in a formalized form.

At the end of the chapter, the steps and problem solutions are systematized into a single matrix, the cells of which specify the application of one of the ML field solutions to the operation of one of the SA steps. This matrix corresponds to the first result of the study—the SA model.

Section 5 reviews articles with research results applicable to the performance of a stage of SA, and describes the ML solutions used in doing so. Section 6 systematizes the articles in the form of a matrix model—according to their relation to a certain SA stage and one of the tasks solved with the help of ML. This matrix corresponds to the second result of the study—a model of ML application to SA.

A conceptual model of a complex SA using ML, based on a hypothetical intelligent framework is described. Section 7 presents the main conclusions of the study and indicates the way forward.

## 2. Research Methodology

The specificity of using SA for IoTS based on ML is as follows.

Internet of Things. IoTS security is of particular relevance, since the disruption of their functioning directly affects the real world—the operation of mechanisms, people’s lives, etc. For example, errors in the operation of smart home security systems can lead to financial losses for owners, incorrect operation of the transport system [15] can lead to accidents and traffic congestion, and errors in the operation of medical IoT devices can lead to the death of patients.

Statical Analysis. The particular difficulty of conducting SA is manifested precisely for IoTS, which have different functional purposes. Thus, devices use various OS, distros and CPU architectures [16]. Each such choice, in particular, is selected based on the tasks of the devices. For example, some IoTS require high operating speed, others demand battery life, others lack ultra-precise complex calculations, etc. As a result, a more generalized and systematized set of SA methods and instruments is needed across the entire IoTS diversity.

The existing SA methods, based in particular on expert-based rules, are being developed for the some type of Desktop PC software. For example, the well-known Hex-Rays decompiler from IDA Pro supports only code for the x86, x64, ARM32, ARM64, and PowerPC processors. Wherein, threat detection based on suspicious network traffic (i.e., through dynamic analysis) will not always reveal a single attack exploiting a 0-day vulnerability. Thus, a security analysis is required even before the devices are actually launched.

System of IoT. The presence of a whole system of interacting IoTS imposes a number of restrictions on how to ensure security. Thus, in particular, dynamic analysis will be of little use, since it is rather difficult to emulate the environment of a device that continuously interacts with the outside world. At the same time, sometimes, security problems manifest themselves precisely in the case of a group of several interacted IoTS and not a single device.

Machine Learning. The large number of IoT manufacturers and the variety of solutions used do not allow the application of SA rules that are created by hand or are required to use manual labor of experts. Otherwise, SA will be extremely resource intensive. A large number of cyber-physical interfaces (sensors, sources of impact, etc.) significantly complicates the development of a test case manually. As a result, a partial replacement of a person’s creative abilities with automation is required. This is what justifies the use of ML.

The first step in verifying applicability of any new set of methods to the tasks at hand should be a review of existing assumptions for such an application. In order to confirm the hypothesis, we formulate an ongoing research problem, compiled from two subtasks:

1. To justify that ML-based solutions can underlie the transformations carried out at each stage of IoTS SA for information security.
2. To make an overview of ML-based solutions, each applicable to each stage of IoTS SA for information security.

The process of solving this hypothesis and its solution is described in this article.

In addition, since the results of the review should give an answer regarding the formulated hypothesis and tasks, the main results of the study will be defined in the following form:

1. The SA model, systematizing in a formalized form the required transformations of IoTS SA stages and possible solutions of ML tasks.
2. A model of ML application for SA, systematizing the existing solutions linking IoTs SA and the possibilities of ML application for its implementation.

**Figure 1:**
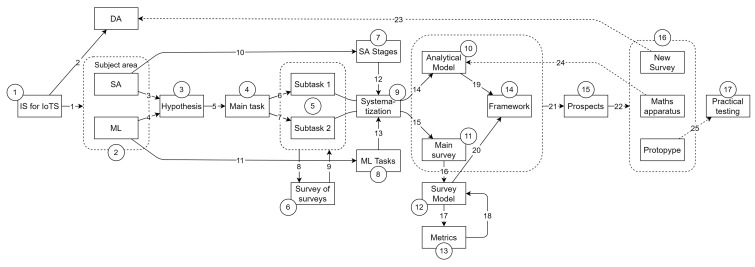
Research scheme.

The obtained results will be useful both in creating a general methodology of intelligent static analysis and in selecting implementations of its specific solutions. In addition, the second result will assess the status and research trends of existing solutions. The research methodology in schematic form, reflecting also its ontology, is presented in Figure 1.

According to Figure 1, the ongoing study has the following layout, in which the actions are marked with the corresponding numbers:

1. Actualization of the research topic as IS of IoTS.
2. Selection of the subject area of the research, as SA from the ML position.
3. Proposal of the hypothesis for confirmation in the study.
4. Statement of the main research problem.
5. Division the main research problem into subtasks.
6. Analysis of publications with existing reviews in the subject area.
7. Allocation and formalization of the SA stages.
8. Allocation and formalization of tasks to be solved with the help of ML.
9. Systematization of SA stages and ML tasks.
10. Building the SA model.
11. Basic overview of the articles in the subject area.
12. Building an ML application model for SA.
13. Obtaining and analyzing publication metrics in the SA subject area.
14. Creation of the intelligent framework concept for SA.
15. Selection of future research directions.
16. Determination of future research perspectives: conducting a similar review for IoTS DA, development of the mathematical apparatus of ML application for SA, creation of a framework prototype.
17. Carrying out practical experiments with the framework prototype and its analysis.

The ontological part of the scheme in Figure 1 is defined by the elements presented on it (in rectangles) and their relationships, with the following values (according to the line numbers):

1. Has current solution paths.
2. Has solution paths not considered in the work.
3. Is the first basis of the hypothesis.
4. Is the second basis of the hypothesis.
5. Sets the main problem of the study.
6. Divides into subtask 1 of the study.
7. Divides into subtask 2 of the study.
8. Explores existing solutions.
9. Concludes that there are no suitable solutions.
10. SA steps are identified and formalized.
11. ML tasks are identified and formalized.
12. Used as the matrix columns in systematization.
13. Used as the matrix rows in systematization.
14. SA model is constructed.
15. Main review of publications is done.
16. ML application model is built for SA.
17. Publication metrics are calculated.
18. Justifies the ML application model.
19. Constructs the framework basis using the solution of subtask 1.
20. Constructs the framework basis using the solution of subtask 2.
21. Predicts continuation of the study.
22. Chooses directions for future research.
23. Analyzes the application of ML to DA.
24. Development of a mathematical apparatus to refine the model.
25. Development and testing of a smart framework prototype.

Note that the Main survey (action 11, Figure 1) is the collection and analysis of publications for each application of the ML tasks for SA stages (it is described in Section 4). This is necessary to collect statistical data on the research history of each application. Statistical data will reveal the main research trends and their possible problems. The general picture of the elaboration of the entire subject area will be visible. At the same time, a large number of publications will be able to confirm the research hypothesis.

The SA stages model (action 7, Figure 1) is needed to create a new model (action 9, Figure 1)—a model applying ML tasks (action 8, Figure 1) for SA stages. The last model is necessary for theoretical confirmation of the hypothesis. In fact, an attempt to create a methodological apparatus for theoretical confirmation of hypotheses of the form “Is it possible to use certain methods to solve requires problems” has been made.

## 3. Analysis of Existing Review Works

### 3.1. ML for SA of IoTs

There are many reviews devoted to collecting, systematizing, and comparing scientific publications on the topic of ML applications for information systems in general and IoTS in particular. However, only a small fraction of them relate to the field of IoTS security, and not, for example, network security. A brief analysis of interesting reviews is given below.

In [8], a comprehensive study of different ways to analyze binary code is presented. A taxonomy of the corresponding framework is given, which consists of four components: feature extraction, feature embedding, analysis techniques, and applications. Greater attention is specifically paid to the application of ML regarding the techniques of code analysis. Thus, there are many works (more than 100) in which ML is applied to the problem of classification, including viruses and their families, string tokens, cryptographic algorithms, and code clones.

Clustering is also described from the following positions, in addition to preparing data for other classifiers: authorship detection, detection of clones, determination of similarity of the program to a virus from the base, and identification of auxiliary features in the code (virus features and entry points in functions). The following works are described concerning the application of ML for binary code analysis: classify malware [17,18,19,20], identify function entry points [21], and recognize functions [22].

Work [7] is devoted to the application of ML exclusively to source code for the following tasks: Recommender Systems—help systems for software developers (code autocompletion, etc.); Inferring Coding Conventions—support of coding style conventions (formatting, variable naming, etc.; Code Defects—detection of anomalies in the code, which can tell you about vulnerabilities; Code Translation, Copying, and Clones—code conversion between different programming languages and search for similar code fragments; Code to Text and Text to Code—conversion between the program code and natural language code; Documentation, Traceability and Information Retrieval—code documentation and searching; and Program Synthesis—mechanisms of “smart” program code generation.

The works [23,24,25,26,27,28,29] describe concerning the application of ML to analyze the source code for anomalies, i.e., code defects. Thus, the review covers only a narrow set of security subdomains.

In the review [9] on software vulnerability analysis and detection, all reviewed works are divided into four categories (with corresponding papers): Vulnerability Prediction Models based on Software Metrics, Anomaly Detection Approaches, Vulnerable Code Pattern Recognition, and miscellaneous Approaches. The first category is devoted to prediction of possible vulnerabilities on the basis of different source metrics [30,31,32,33,34,35,36,37,38] and binary code [37,39], as well as code commits (which goes beyond SA).

The second category is devoted to detecting anomalies in the code, which may be a consequence of the vulnerabilities in it. This is done by looking for deviations in API calls from applicable patterns [40,41,42,43,44,45,46] and missing typical checks [45,47,48]. The third category is devoted to the direct detection of vulnerabilities in code by pretrained patterns. For this purpose, the works [49,50,51,52,53,54,55,56,57,58] apply classification and in [52,56] also clustering. The fourth category contains descriptions of approaches not included in the previous three categories.

The category mentions the following works and their ideas: fuzzy testing using genetic algorithms [59]; bug report analysis using classification to identify hidden vulnerabilities [60,61]; searching for memory corruption vulnerabilities by source code using genetic algorithms and Fish School Search [62]; classification and prediction of false positives in SA vulnerabilities in Web application code and automatic fixing them [63]; and improving SA efficiency and scalability for software repositories [64]. Thus, although the research in the fourth category is related to SA, it is rather of an auxiliary nature and will not be discussed further.

Work [65] focuses on the security of software interactions in complex information systems through visualization. In particular, to increase the intellectualization of the analysis of interactions, it is proposed to use classification, anomaly detection, clustering, regression, and dimensionality reduction.

As we can clearly see, despite the vast number of works, most of them are applicable only to the source code and have far from always satisfactory efficiency. The review concludes that the field of detection of vulnerabilities in code using ML is insufficiently developed. In spite of the rather extensive reviews (by the number of publications reviewed—from 100 to 200), all the works considered in them can be attributed only to direct software security testing.

However, the processes of collecting such a code, its preparation and presentation to an expert are of a partial nature, not being part of a unified static analysis methodology. As a result, it is not always possible to build a static analysis process out of several coordinated steps. It is necessary to pay attention to these questions, which will be done in this article.

### 3.2. ML for IoT Security

We also analyze the latest works devoted to other reviews on the topic of using ML to enhance IoT security.

The work [66] is devoted to the problem of identifying IoT devices. The article provides an overview of identification methods based on ML. Identification mechanisms are separated into four groups: pattern recognition, deep learning identification, unsupervised learning identification, and anomalous device detection. Thus, this work is partially related to the current subject area. However, the proposed solutions can only be attributed to SA Stage 1.

In [67], the security trends for IoT devices are assessed. A total of 20 surveys are reviewed, and ML is considered in only 25% of them. The main taxonometries, however, relate to the identification of groups of attacks and not how to defend against them. The use of ML to improve security is mainly considered in the context of identifying the dynamic attacks, and not static weaknesses in the implementation of devices (i.e., in program code, algorithms, architecture, etc.).

The work [16] emphasizes the relevance and underdevelopment of solutions for the search for malicious IoT software. Particular attention is paid to the executed Unux-like programs in the ELF format. The statistics show that the OS for IoT is 70% Linux (file format—ELF), and Windows—22% (file format—PE); the frequency of using other operating systems is less than 20%.

The main fields of the ELF header are considered. A feature of the survey is the consideration of approaches to searching for malicious code from the standpoint of their independence from the architecture of the program execution. A taxonomy of features obtained from ELF programs is created, which can be used for static and dynamic analysis to search for malicious code in IoT devices.

The taxonomy separates features into the following: for SA—metrics, graphs and trees, sequences, and dependencies; for DA—logs and resource usage. Despite some closeness of this survey to the current research, its results can be partially attributed to Stage 3 only (with partial consideration of Stage 1 and Stage 2), i.e., not to the full SA process. At the same time, only such ML problems as classification (and its special case—detection) are highlighted.

In [68], the IoT threats as well as ML-based IoT security models are outlined. A layered IoT model is proposed. A Q work flow of threat detection is described based on this model. However, all countermeasures involve analyzing the network activity of devices at various network levels, i.e., they belong to dynamic analysis. At the same time, there is no direct analysis of IoTS as a software system. From ML techniques, only classification, clustering and regression are indicated. Techniques, such as rule-based systems and reinforcement learning, are highlighted separately.

In [69], the application of Federated Learning (FT) for IoT is considered. As a result of using this technique, there will be no need to transfer data for training. The FL-IoT system is presented, describing local training, FT-server and exchange processes between them. The possibility of using FT also for detecting malicious software in a static way is indicated. The main applicability of ML is in classification, regression, and anomaly detection. The main countermeasures for IoT threats are through dynamic device analysis. At the same time, the survey is devoted specifically to the implementation of FT in IoT and not to systematize SA. Note that the use of FT can increase efficiency and safety when conducting the SA (at all stages and tasks of ML).

The work [70] is devoted to IoT security in 5G networks, characterized by a huge number of devices and high data transfer rates. The existing authentication schemes at the physical layer are analyzed. Schemes are considered from the perspective of applying ML. ML technologies, such as autoregressive random process, Kalman filtering prediction, reinforcement learning (specifically Q-learning and Dyna-Q), AdaBoost classifier, and kernel machine, are investigated.

The work [71] emphasizes the relevance of IoTS security due to their wireless transmission network, large coverage area, heterogeneity of services provided, and cyber-physicality. The application of ML methods for creating attack detection models is analyzed. It mainly describes the application of deep-learning-based classification.

The work [72] focuses on securing the IoT using ML. Four types of defenses are considered: device authentication, DoS and DDoS attacks defense, intrusion detection, and malware detection (relevant in the context of current research). The possibilities of using ML based on the following models are indicated: convolutional neural networks, SVM, BP neural networks, and ensemble learning.

The work [73] provides an overview of using honeypots to collect information about malware that attacks PE files, data streams, and IoT. A feature of the considered honeypots is the use of ML for data collection and analysis. Based on a “raw” review of articles in the IEEE Xplore and ACM databases, it is concluded that there is an increasing trend in the use of such smart honeypots. However, there is no specificity regarding the details of the use of ML.

The work [74] provides an overview of ways to detect malware for Android systems (including those in the IoT field) using ML. Experiments were carried out using the Naive Bayes, J48, Random Forest, Multiclass Classifier, and Multi class perceptron algorithms. The main application of ML here is to solve a classification problem.

Based on this review of other surveys on the application of ML for IoT security, the following conclusions can be drawn.

First, the considered solutions mainly belong to the field of dynamic analysis, since they process external effects of malicious devices during their operation. In contrast, our review focuses to SA. Such an analysis can be carried out both without the direct launch of IoTS, and only at a certain point in time.

Second, the solutions reviewed mainly seek to introduce taxonometry for systematizing devices, offering countermeasures based in part on ML. Our review introduces the systematization of all SA stages using basic ML tasks. We investigate not a problem of identifying threats, but a problem of identifying ways to counter threats during SA.

Third, the considered solutions mainly conducted systematization according to existing threats and solutions. This can lead to the fact that hypothetically some of the elements will be lost (for example, if such solutions have not yet been proposed). We initially synthesize the entire set of solutions—as a 4 × 5 matrix (or most of it). Then, we prove that every such decision has a right to exist. Thus, our approach is more methodologically correct.

Fourth, none of the reviews considered the entire set of main tasks solved with the help of ML—classification, anomaly detection, regression, clustering, and generalization. As a rule, IoT security applies only the solution of the first two problems. SA of a binary code is done basically only with the use of classifiers. Thus, new applications of more rare solutions in ML for the IoT field can be “missed”.

Fifth, the considered solutions are mainly aimed at studying individual IoT devices or their interactions. We position the review as an analysis of the whole IoTS. In the interests of this, the concept of Intelligent Framework is proposed.

## 4. SA Model

To create models of the subject area, we consider in more detail the SA area from the position of application of ML in it. To do this, we use both known theoretical preconditions and practical experience in the published studies. First, the whole SA is heterogeneous (and often not linear), which means that it can be divided into certain time segments or stages (with a certain purpose). Secondly, the solutions must use ML. Consequently, it is possible to distinguish the tasks for which ML is applicable. As a result, it is possible to systematize in a single model both the stages of SA and the ML solutions applicable to them.

For the stages, classical and generalized time segments were used (excluding, for example, balancing and augmentation).

The purpose of the Model is as follows. First, its formation itself formally confirms the possibility of applying various solutions to ML tasks at each stage of SA. At the same time, such a proof will be built on a single basis (using Form and Content, described below). Thus, the module serves as a theoretical proof of the Hypothesis. Secondly, the theoretical model can be used for practical implementations of algorithms for solving problems for stages.

### 4.1. Stages of Static Analysis

Since SA is a complex process consisting of an entire set of tasks to be solved and using qualitatively different data, it is usually divided into several stages. For convenience, we use the following formalization to assign each of the activities to the stages of SA. Any data is represented by the information it contains—Content (*C*), and appearance—Form (*F*). For example, a line-by-line log of execution errors consists of: Content—errors and Form—text strings.

Then, certain data, both stored in the IoTS and transformed in the SA process, can be written as a tuple <F∣C>. From this position, we consider each stage as a Content and Form transformation of incoming data. Then, the formalized version of the transformations of the stages can be written as:

(1) $$<F_{i+t}|C_{i+1}>=Stage_{i}\left(<F_{i}|C_{i}>\right),$$

where $F_{i}$ and $F_{i+1}$ is Form before and after *i*-th Stage, $C_{i}$, and $C_{i+1}$ is Content before and after *i*-th Stage, $Stage_{i}\left(\right)$ is a transformation of *i*-th Stage. Summarizing the described approaches and tools for SA software [75,76,77,78], we can combine them based on the method of data transformation. With a sufficient degree of abstraction, without violating the correctness, we can distinguish the following four stages of IoTS analysis.

#### 4.1.1. Stage 1. Data Collection

This is the initial stage and is intended to isolate from the IoTS that subsystem (“vertical component”) or level (“horizontal component”), which will be further processed. This step is necessary because the IoTS, like any complex and multifaceted object, can be considered in terms of different aspects. A specific aspect must be chosen at this stage. The input of the stage is the information about the IoTS itself. The output of the stage receives only part of the information about the IoTS selected for processing.

The formal notation of Stage 1 is as follows:

(2) $$\left\{\begin{array}{c} <F_{2}|C_{2}>=Stage_{1}\left(<F_{1}|C_{1}>\right)\\ F_{2}=F_{1}\\ |C_{2}|<|C_{1}| \end{array}\right.,$$

where operator ∣X∣ is a set power calculation, which together with the third equation means that after Stage 2 the Content size has decreased. This is explained by the fact that at this stage only part of IoTS information ($C_{2}$) is selected, and not information about the entire IoTS ($C_{1}$). At the same time, the IoTS Data Form does not change at this stage, which is shown in Equation (2).

This step may not be explicitly related to IoTS tasks, although it is necessary. Typical actions for the stage are the following: selecting the direction and level of research; unpacking data containers (archives); typing and attributing files; structuring executable code architectures, etc. For example, at this stage, only executable files (PE format for Windows or ELF format for Linux) will be selected from all IoTS files.

#### 4.1.2. Stage 2. Data Preparation

This stage is designed to convert the data about a part of the IoTS to a form suitable for the processing methods. Without this step, all processing methods would have to adapt to the form of the data in the IoTS, which is naturally not productive. The input of the stage is the IoTS piece of information selected in Stage 1. The output of the stage creates part of the IoTS information converted into a suitable form. Naturally, if there is more than one processing method, this step must convert the IoTS information into a form suitable for each method. Thus, the step may separate the Form and Content population for each method.

The formal notation of Stage 2 is as follows:

(3) $$\left\{\begin{array}{c} <F_{3}|C_{3}{>}^{j}=Stage_{2}^{j}\left(<F_{2}|C_{2}>\right)\\ F_{3}\neq F_{2}\\ \underset{j=1..N}{⋃}C_{3}^{j}\equiv C_{2} \end{array}\right.,$$

where $<F_{3}|C_{3}{>}^{j}$ is the Form and Content after Stage 2 suitable for the *j*-th processing method; $Stage_{2}^{j}\left(\right)$ is the part of Stage 2 where the data after Stage 1 is converted to the form suitable for each *j*-th method; and operator “≡” is identity, which together with the second equation means that, after Stage 3, the total Content has not changed (N is the number of processing methods); $\underset{j}{⋃}C_{j}$—merging of all Content. The second equation is explained by the fact that this stage “tweaked” only individual Form parts of Content, without changing the total information in Content. The fact of the change of the Form in the step is reflected in the second equation.

This phase does not solve specific IS problems but converts the data into a form that best represents the features associated with such problems. Typical actions for this stage are: creating a project and environment; separating IoTS modules; obtaining binary code sections; disassembling; separating subprograms in the code; building an Abstract Syntax Tree, Intermediate Representation Tree, Call Tree, Control Flow, Data Flow, etc. For example, at this stage, for each IoTS file will be built a graph of the execution of instructions of its functions—Control Flow, as well as calls of other functions from them—Call Flow.

#### 4.1.3. Stage 3. Data Processing

This stage is the main and most difficult both from a theoretical and practical point of view. At this stage, all the methods of data processing are performed in relation to IS (vulnerabilities, malwares, stego, bugs, backdoors, etc.). The results of this stage are determined by the combined results of all its methods. At the input of the stage comes the data, prepared for its work with the help of Stage 2. Each method, as a rule, consists of many complex algorithms, often creating qualitatively new data sets. Consequently, at the output of the stage, we can obtain data with completely different Form and Content. The formal notation of Step 3 is as follows (for one method):

(4) $$\left\{\begin{array}{c} <F_{4}|C_{4}>=Stage_{3}\left(<F_{3}|C_{3}>\right)\\ F_{4}\neq F_{3}\\ C_{4}\neq C_{3} \end{array}\right.,$$

where $Stage_{3}\left(\right)$ is the part of Stage 3 corresponding to one of the methods for which data were generated in Stage 2. As indicated, the resulting Form and Content can have significant variations, as reflected in Equations (2) and (3).

At this stage, IS tasks are solved directly: vulnerabilities are discovered, virus clones are found, code security metrics are evaluated, code vulnerabilities are neutralized, predictions are made regarding future threats, etc. Typical actions at this stage are: creating and updating an infiltrator model; searching for and predicting vulnerabilities; searching for clones; converting code to a human-oriented form; authorship determination, etc. For example, at the stage by the aggregate of function calls (received by Control Flow and Call Flow) this function can be detected as malicious. The exact match between the function instructions and the malware from the database is not necessary.

#### 4.1.4. Stage 4. Result Formation

Result formation is the final stage, providing all the results of the IoTS analysis. At this stage, the data obtained during processing at Stage 3 are converted into a single form (less often, into their set), ready for further analysis—both human and software. At the input of the stage are the results of all methods of data processing. The output of the stage can be considered problem-oriented, depending on the purpose of the analysis application.

The formal notation of Stage 4 is as follows:

(5) $$\left\{\begin{array}{c} <F_{5}|C_{5}>=Stage_{4}\left(\underset{j=1..M}{⋃}<F_{4}|C_{4}{>}^{j}\right)\\ F_{5}\neq F_{4}\\ C_{5}=\underset{j=1..M}{⋃}C_{4}^{j} \end{array}\right.,$$

where $Stage_{4}\left(\right)$ is the transformation of Stage 4, taking as input all the set of methods results (by number *M*). The form of data is likely to change after applying stage (although, theoretically, it can remain the same for trivial problems). The Content after the stage will consist of all the Content obtained by the methods at the previous stage.

#### 4.1.5. Form and Content Transformation

The stage adapts results of IS tasks to the required view given in the specific SA goal. Typical actions for the stage are as follows: systematization of parameters and characteristics of IoTS objects; classification of viruses and vulnerabilities; displaying the list of found viruses and vulnerabilities; displaying security metrics and statistics; selection of IS recommendations, etc. For example, at the stage on the obtained list of malicious functions, the output of this stage may consist of the list itself as infection statistics can. For an easier presentation of stages analyzing the IoTS, we present their Form and Content transformations in Table 1.

The formalization from Table 1 will be used implicitly when assigning each of the following activities to the SA stages.

### 4.2. Tasks Solved by ML

Now, we consider the problems usually solved with ML. According to both general theory and a large number of scientific papers and their reviews ([79,80]), all ML problems can be divided into the following.

**Classification** is assigning objects to a given class. The application requires teacher-led training regarding the features of each class. One of the most common applications in IS is the division of program code into safe and malware, using the features of the latter.

The formal notation of a solution to the classification problem is as follows:

(6) $$\left\{\begin{array}{c} \left\{O\right\}\to {\left\{O\right\}}^{T_{1}}...{\left\{O\right\}}^{T_{N}}\\ i\neq j:{\left\{O\right\}}^{T_{i}}\cap {\left\{O\right\}}^{T_{j}}=\emptyset \\ Given:\{O_{Train}^{i}\in {\left\{O\right\}}^{T_{k}}\} \end{array}\right.,$$

where {O} is the set of classified objects; ${\left\{O\right\}}^{T_{i}}$ is the set of objects belonging only to class $T_{i}$ (following the second equation of the formula, the intersection of the set of objects of different classes equals an empty set “∅”); Given is the indication of given solution parameters as a set of predefined training objects $O_{Train}^{i}$, assigned to the corresponding classes $T_{k}$.

**Anomaly detection** is the assignment of objects to a new unknown class, thus, distinguishing the task from classification. Applications are possible both with and without training with the teacher—on normal and anomalous data—on deviations from normal data. One of the most frequent applications in the field of IS (besides the typical detection of network attacks) can be the detection of differences in the operation of the program from normal (function calls, disk operation, processor power consumption, etc.), which often indicates the presence of malware in it.

The formal notation of a solution to the anomaly detection problem is as follows:

(7) $$\left\{\begin{array}{c} \left\{O\right\}\to {\left\{O\right\}}^{N},{\left\{O\right\}}^{A}\\ O^{i}\in {\left\{O\right\}}^{N},O^{j}\in {\left\{O\right\}}^{A},O^{i}\approx O^{j}\\ Given:\emptyset \|\left\{O_{Train}^{i}\in {\left\{O\right\}}^{N}\right\},\left\{O_{Train}^{i}\in {\left\{O\right\}}^{A}\right\} \end{array}\right.,$$

where {O} is a set of objects to detect anomalies; ${\left\{O\right\}}^{N}$ is a set of normal objects; ${\left\{O\right\}}^{A}$ is a set of anomalous objects significantly different from normal (which is shown by the second equation, the symbol “≈” is used for significant difference); Given is an indication of given solution parameters as two options (through operator OR—“‖”): None (*∅*) or a set of pre-known training objects ($O_{Train}^{i}$ and $O_{Train}^{j}$), assigned to normal (${\left\{O\right\}}^{N}$) and abnormal (${\left\{O\right\}}^{A}$). Thus, the first part of the last equation is true in the case of detection without a teacher, and the second part is true with a teacher.

**Regression** is the prediction of a continuous value for datasets (as opposed to classification, where a discrete value—a class—is determined). Similarly to classification, the application requires training with a teacher. In IS, it can be used to predict errors in future programs.

The formal notation of a solution to the regression problem is as follows:

(8) $$\left\{\begin{array}{c} \left\{O\}\to \{D\right\}\\ D^{i}=F\left(O^{i}\right)\\ Given:\{F\left(O_{Train}^{i}\right)=D^{i}\} \end{array}\right.,$$

where {O} is the set of objects to determine the regression; {D} is the set of numbers from the set of real “R”, matched to objects {O}; $D^{i}$ is *i*-th number matched to *i*-th object $O^{i}$ using some function F(); Given is the indication of given solution parameters as a set of predefined objects $O_{Train}^{i}$, matched (using function F()) to the corresponding numbers $D^{i}$.

**Clustering** is the division into groups according to their attributes. Application is possible without a teacher, because the classification looks for internal patterns in the data. Generally, clustering is applied before other classifications. However, in the IS field, it can be applied to search for malware clones in the code of the IoTS objects under study.

The formal notation of a solution to the classification problem is as follows:

(9) $$\left\{\begin{array}{c} \left\{O\right\}\to {\left\{O\right\}}^{K_{1}}...{\left\{O\right\}}^{K_{N}}\\ i\neq j:{\left\{O\right\}}^{K_{i}}\cap {\left\{O\right\}}^{K_{j}}=\emptyset \\ Given:N \end{array}\right.,$$

where {O} is the set of clustered objects; ${\left\{O\right\}}^{K_{i}}$ is the set of objects belonging only to cluster $K_{i}$ (following the second equation of the formula, the intersection of the objects set from different clusters equals an empty set); and Given is an indication of the given solution parameters as their absence (∅).

**Generalization** (dimensionality reduction) is transformation of a feature space of one dimension into a space of smaller dimension with minimal loss of contained information. For reasons similar to clustering, the application of regression is possible with learning without training. Similar to clustering, generalization is applied as preprocessing of data before other classifications. In the IS field, dimensionality reduction can also be used for auxiliary tasks related to threat mapping and selection of protection recommendations.

The formal notation of a solution to the generalization problem is as follows:

(10) $$\left\{\begin{array}{c} \left\{O∼X\}\to \{O∼Y\right\}\\ X^{i}={\left\{x_{k}^{i}\right\}}^{k=1...N}\\ Y^{i}={\left\{y_{k}^{i}\right\}}^{k=1...M}\\ N>M\\ Given:\emptyset \end{array}\right.,$$

where {O} is a set of objects described (using operator “∼”) by feature set {X} of dimension *N*; {Y} is a set of features of objects with less dimension *M* than the original; $x_{k}^{i}$ and $y_{k}^{i}$ is a value of individual *k*-th feature for *i*-th object; and Given is an indication of given solution parameters as number of clusters, by which the original objects will be distributed.

### 4.3. SA Model Representation

The main stages of SA (Section 3.1) and the main tasks to be solved in ML (Section 3.2) were specified earlier. Furthermore, a formalized description of stages and tasks was given. Thus, the previously established hypothesis regarding the application of ML to SA can be transformed to verify that each of the stages of SA can be based on the solution of one of the ML tasks.

We represent this assumption in the form of a matrix model, where the columns are SA stages, the rows are ML tasks, and the cells are a formalized record of stage actions based on the solution of one of the ML tasks. The possibility of filling all the cells of the matrix of such a model will mean a theoretical confirmation of the hypothesis. The analytical model of ML solutions for the SA stages is presented in Table 2.

Each cell of Table 2 represents an ML statement for the stage operation, taking a tuple from Form and Content input data (obtained from the IoTS or previous) and returning the same tuple from the output data (produced by the current stage). To simplify the record from the formal entries of ML problem solutions, we use only the first line describing the main action, thereby, omitting the rest, indicating the conditions on the input and output variables.

There will be preceding and succeeding non-ML statements in addition to the above-mentioned ML statements, since in addition to the intelligent component, each step performs strictly defined rules (e.g., unpacking archives with files, ranking documents by their size, searching for malicious code areas in databases with signatures, translating tabular data into the form of graphs, etc.).

Expert analysis of the matrix (see Table 2) allows us to conclude that the cells of the matrix are written correctly. Hence, each of the ML problem solutions can be the basis for transformations of each of the ML stages. Thus, the first subtask can be considered solved, which confirms the main hypothesis of the current scientific research from the theoretical point of view.

When conducting an expert analysis, 10 specialists were selected, meeting the following requirements:

- PhD for more than 5 years,
- acquaintance and application in practice of system analysis,
- knowledge of the basics of machine learning,
- experience in the field of the IoT security,
- analytical modeling skills,
- lack of explicit affiliation with the authors of the current investigation, and
- general competence in the subject area.

Then, each selected expert was introduced to the specifics and conducting SA Steps as well as with the goal to create an analytical model of CA using ML. Each expert was presented with an analytical model in matrix form to study (see Table 2). The expert conducted an individual analysis of the resulting analytical model. Then, a survey was conducted with the experts regarding each cell of the table (4Stages×5Tasks=20cells) on the following five questions:

1. Do you understand the meaning of the entry?
2. Is the formal notation correct?
3. Does the entry match this SA Stage (column heading)?
4. Does the record correspond to the given ML Task (row header)?
5. To what extent the receipt of the record of the current stage logically follows from the record of the previous stage (except for Stage 1)?

The experts evaluated the model (i.e., answers to questions) using a point system, from 1—the answer is completely negative, to 5—the answer is completely positive.

Then, the answers for each cell of the table were summarized and averaged. The distribution of average responses across all cells ranged from 4.5 to 5 points. This result confirms the correctness of the obtained analytical model.

## 5. Systematization of SA Stages and ML Solutions

Based on the task, we present the model that systematizes the existing research in the form of the following table: columns are represented by stages of IoTS analysis; rows—tasks solved using ML. Next, we consider the research papers applicable to IoTS analysis. We assign each work by its attributes to both one (or several) stages and one (or several) ML tasks. Thus, the entire set of such studies should hypothetically allow us to fill all the cells of the table.

We introduce the notation work groups located in cells as follows—Sx_Ty, where x is the number of analysis stage (x=1...4), y is the number of ML task (y=1...5). For example, if a scientific paper outlines the process of searching for vulnerabilities in code (Stage 3) through classification (Task 1), then it belongs to the group S3_T1.

A large number of papers were considered for the following reasons.

First, the presence of more than a single study on the application of the solution of each ML problem at each stage of the SA will be considered more reliable evidence of the hypothesis.

Secondly, it is necessary to determine the presence and degree of research of each stage of the SA from the standpoint of solving each task of the MO. This will allow one to identify the most unaffected areas (compared to the rest) and pay special attention to them.

Thirdly, the statistical distribution of the characteristics of work (for example, the stage of SA, the task of MO, bringing to the experiment) over the years will make it possible to predict the success of each affected area.

It is most difficult to divide into groups S2_T5 and S3_T1, since data is often prepared by downsizing before the typical classification. We further divide the works into these groups based on whether the data after downgrading can be used in isolation (group S2_T5) or whether they are for training only (group S3_T1). Additionally, we group all the works considered further by the SA stages.

### 5.1. Stage 1. Data Collection

The authors of this paper proposed and tested the typing of basic information systems files, including IoTS (with the extensions .exe, .py, .doc, .png, .txt, .c, and .cpp) [81], and binary code processor architectures (amd64, arm64, armel, armhf, i386, mips, mips64el, mipsel, ppc64el, and s390x) [82,83,84], using ML classifiers. These works belong to the S1_T1 group.

Similarly to papers [81,82,83,84,85] applied an SVM (Support Vector Machine) to classify files by blocks in the file system, which may be needed in forensics. The work belongs to the S1_T1 group.

An earlier work [86] solved the problem of preprocessing data files without any description, that are the result of events such as System Crash, data interception by secret services, storing information in “impersonal” files to prevent leaks, etc. For this purpose, similarities between files are sought by creating clusters of their attributes. In [87], a classical application of clustering for grouping text documents using the TF-IDF (Term Frequency—Inverse Document Frequency) scheme is described.

This approach can be applied to the initial collection of documents. All of them will have common features and, therefore, will be investigated by similar methods. Similarly, the work [88] discusses clustering algorithms (K-means, K-medoids, Single Link, Complete Link, and Average Link) for preparing files for forensic processing by experts of the corresponding field. The work belongs to the S1_T4 group.

In [89], a method for searching and localizing signatures and machine-printed text in images using classification is described. MIL (Multiple Instance Learning) is used for this purpose. The method is not directly related to IS but can be used for document indexing in forensic and business fields (as in [90]). The work belongs to the group S1_T1. In [91], MIL is used for clustering. Thus, the work can be classified as S1_T4.

In [92], a classical method for identifying packed executable files (PE format) using the SVM classifier is described. This makes it possible to define objects to be first unpacked and then processed. The work belongs to the S1_T1 group.

In [93], a method for identifying documents containing packed executables is described. In contrast to [92], an anomaly detection method is used for this purpose. Thus, the work belongs to the S1_T2 group.

The work [94] indicates the need to identify anomalies in file integration systems. The proposed solution is based on the principles of self-learning. The work belongs to the S1_T2 group.

In [95], the applied problem of file optimization is solved by predicting the lifetime of files by absolute path symbols. By lifetime, we mean the interval between file creation and the last reading. Regression methods based on Random Forest and Convolutional Neural Network Model are used for prediction. The method can be used to sort files by lifetime and select for processing those that are within a given range. This can be used in forensics to establish files related to the chronology of cybercrime. Thus, the work belongs to the S1_T3 group.

In [96], the classical problem of stego attachment detection is solved. For this purpose, a universal solution based on Multiple Linear Regression is proposed. Additionally, the length of the nested message is obtained. The images found in this way can be subjected to additional processing using the revealed information. Thus, the work belongs to the S1_T3 group and, to some extent, to the S2_T3 group as well (due to the partial localization of the stego field).

In [97], the classical method of document classification based on SVM with prior dimensionality reduction using PCA (Principal Component Analysis) is described. The work belongs to the S1_T1 group.

In [98], a method to recognize handwritten inscriptions in graphical images is investigated. A convolutional and recurrent neural network is used for this purpose. The inscriptions may contain sensitive information (e.g., passwords or personal data) and, therefore, their detection and verification can be applied in the interest of IS. In addition to the fact that a handwritten inscription is present, its translation into a specific text is performed and hence the work can be classified as S1_T1 and S2_T1 groups.

The work [99] deals with enhancing activities in forensics. For this purpose, it is proposed to use machine learning in terms of classification and clustering to process documents and cell phone applications. The authors propose to apply SVM and kNN for this purpose. Thus, the work belongs to groups S1_T1 and S1_T4.

### 5.2. Stage 2. Data Preparation

According to the reviewed review [8], the works [21] and [22] can be classified as S2_T1.

In [100], the secondary problem of decompilation is solved—determination of variable types. For this purpose, it is proposed to use the SVM and Random Free classifiers, which showed better results in comparison with others. The work belongs to the group S2_T1.

In [101], an ML-based classification method is described, which allows to determine Function Entry Points (the starting byte of each function) with high accuracy. This is necessary for disassembling function instructions in the interest of further analysis. The work belongs to the S2_T1 group.

The review [102] discusses the evolution of architecture reconstruction methods. It contains discussion and references to works which use clustering for reconstruction of program architecture. In the future, the obtained architecture can be processed for the detection of high-level vulnerabilities. The work belongs to the S2_T4 group.

The work [103] solves the problem of software refactoring. It is proposed to apply the HASP clustering algorithm to group software classes into packages.

Work [104] is devoted to a new approach for recovering binary malware code running on embedded devices considered by Sykipot on Smart cards. This is done by collecting data from the side channels concerning the power consumption of the device. PCA and LDA (Linear Discriminant Analysis) methods are used to reduce the dimensionality of the feature space and then kNN (K-Nearest Neighbors) classification is applied. It is shown that this technique can be used in practice, not only in theory. This work is similar to that described in [105]. The work can equally be attributed to the S2_T5 and S3_T5 groups.

In [106], a sequence of executable binary instructions is converted into a grayscale image. Dimensionality reduction by LDA is then applied, both to reduce the image and to obtain a more optimal training sample. Thus, the work belongs to the groups adjacent with respect to Stage 3: S2_T5 and S4_T5.

In [107], a method built on a logistic-regression classifier that predicts row and column separators in tabular data is proposed. This task can be common when preparing data in files for processing by the methods in Stage 3. Thus, the work belongs to the S2_T3 group.

In [108], a method based on deep neural networks is described that aims at analyzing log text. The anomalies detected in the text will carry the most useful information about application startup failures. In the interest of SA, this can be used to isolate large data files of the most suspicious information: for example, the failure of critical services in the IS. The application of the method will help form more substantial results in Stage 4. Thus, the work belongs to groups S2_T2 and S4_T2.

Similar to [108], the article [109] describes the experiments to identify anomalies in the system logs of OpenStack. For this, a SVM with different cores is used. The work belongs to the S4_T2 group.

The previously described work [96] belongs to group S2_T3, and work [98] belongs to group S2_T1.

### 5.3. Stage 3. Data Processing

According to the examined review [8], the following works can be classified as S3_T1: [17,18,19,20] since they detect vulnerabilities by means of classification. We also mention a work [58] that applies regression to predict vulnerabilities in new Test Cases on existing ones for one set of programs. The work can be classified as S3_T3.

According to review [7], the works [23,24,25,26,27,28,29] detect anomalies in the code and belong to group S3_T2.

According to review [9] works [49,50,51,52,53,54,55,56,57,58] belong to group S3_T1, works [40,41,42,43,44,45,46,47,48] are S3_T2, works [30,31,32,33,34,35,36,37,38,39] are S3_T3 and works [52,56] are S3_T4.

The work [110] is devoted to the detection of malicious code in software using machine learning. Taxonometry is introduced, highlighting such process steps as the presentation of the file with the code, the identification of signs and the direct classification of the malicious code. At the last step, classifiers, such as Artificial Neural Networks, Bayesian Network, Naive Bayes, Decision Trees, kNN, Boosted Algorithms, SVM, Voting Feature Intervals, and OneR are applied. The work belongs to the S3_T1 group.

In [111], a text categorization approach is applied to n-grams of binary program code. Various classification methods are then applied to categorize the sample as safe or malicious software. The work belongs to the group S3_T1.

In [112], we describe the application of classification based on a neural network (with layers: input, embedding, bidirectional long-term memory, attention, and output) trained on the binary code of known vulnerabilities (from NVD and CWE databases). The application of the method to real VLC and LibTIFF software, often used in IoT devices, is shown. The work should be classified as S3_T1.

In [113], the developed software HOSTBAD (Host-based Anomaly Detection) for detecting malicious Android applications is described. It solves the task of detecting anomalies with ML using the features: received/sent SMS, received/sent calls, device activity state, and running applications/processes. A solution similar to [113] was proposed in [114] but was based on a DCA (Dendritic Cell Algorithm). Both works belong to the S3_T2 group.

In [115], an approach for malware classification in Android applications is described, the main one being ensembles of methods, including T-SNE (t-Distributed Stochastic Neighbor Embedding). Although T-SNE is mainly used to visualize data by downsizing the space (to 2D or 3D), in this case, it provides significant assistance to other ensemble classifiers: Gradient Boost Decision Tree, k-NN, Extra-Trees, Logistic Regression, and Neural Network. As a result, it provides more accurate detection of malware. Thus, the work belongs to the group S3_T5.

In [116], various binary data feature detection methods (CFsSubset, Principal Components, InfoGainAttribute, Correlation AttributeEval, GainRatioAttribute, and SymmetricalUncertAttribute) based on n-grams are discussed and then used for classification and malware detection. The best results are achieved by applying PCA and SVM. The work can be equally attributed to groups S3_T1 and S3_T5.

For applications requiring user permission, [117] discusses the method of malware detection as follows. First, the permissions dimensionality of the Android application is lowered using PCA. Second, the SVM classifier is applied to detect malware. The work can be attributed equally to groups S3_T1 and S3_T5.

In [118], the possibility of counteracting attacks using ML without the direct usage of files is discussed. For this purpose, Perceptron is used to detect anomalies in the command lines of standard Windows operating system utilities. The work belongs to the S3_T2 group.

In [119], a way to detect malware in PDF (Portable Document Format) files is described. PCA and the artificial neural network are used for this purpose. The work belongs to the group S3_T1.

In [120], it is proposed to visualize malicious Android applications and then classify the obtained images. SVM, KNN, and Random Forest are used for this purpose. The work belongs to the S3_T1 group.

Work [121] is devoted to the security of IoT devices with command line interpreters typical for Linux shells. It is assumed that malicious software, using shell commands, can perform both system hacking and further infection. The proposed solution is the ShellCore software solution based on static code analysis detecting the malware by its use of shell commands. The solution is based on classification, which allows assigning the work to groups S3_T1 and S3_T5.

The previously described works [104,105] belong to the S3_T5 group.

### 5.4. Stage 4. Result Formation

In [122], an o-glasses method is proposed, which visualizes document files (not necessarily executable) to search for shellcode in them. A high F-measure is claimed (about 99.95 percent) for ×86 binary code. A special one-dimensional convolutional neural network (1d-CNN) is used for this purpose. Although the method implements the full cycle of code analysis, it solves the task of malware visualization. Therefore, the work should be classified as S4_T1.

The work [123] deals with two problems related to the detection of malware: (1) the detection of malware signatures from logs (e.g., the xml created when executing an .exe file in a sandbox) for further training of classifiers and (2) the precise detection of groups of mutant malware. To solve the first problem, ensembles of classifiers are proposed. To solve the second problem, clustering is proposed. To visualize the obtained results in 2D space, an algorithm for decreasing the dimensionality of t-SNE (t-distributed Stochastic Neighbor Embedding) space is used. Thus, the two approaches to solving problems, as well as the way to visualize the malware, refer the work to the groups S4_1, S4_4 and S4_5, respectively.

The work [124] addresses the issue of effective interaction between visualization methods and anomaly detection methods (as outlier). For this purpose, the author’s algorithm «hdoutliers», different from special methods (described in many articles [125,126,127], etc.), is proposed. Thus, the work belongs to the S4_T2 group.

In [128], a method for detecting anomalies in the system log containing both natural language text and numerical values is proposed. This can be applied in the last stage of SA for the data collected by the methods in Stage 3. Therefore, the work belongs to the S4_T2 group.

In [129], a method for automatically classifying vulnerabilities by their textual description using machine learning is described. Such a method can be applied to the detected vulnerabilities at the SA result formatting stage so that they are better presented to the expert, which places this work in the S4_T1 group.

In [130], an attempt is made to predict 0-day vulnerabilities in products based on vulnerabilities contained in NVDs. Linear and quadratic regression models are used for this purpose. Clearly, the method can be applied to predict new IP vulnerabilities based on those found (in Stage 3). Thus, the work belongs to the S4_T3 group.

The work [131] evaluates the influence of the depth of field of the image on the subjective “attractiveness” and image quality. Using logical regression and a deep neural network, it is possible to predict the attractiveness of an image by its quality. A number of experiments were performed. Thus, it is possible to adapt the methods of visualizing the results of SA for further processing. The work belongs to the S4_T3 group.

A solution similar to [129], but which classifies Transport Infrastructure of a Smart City threats, is described in [132]. For this purpose, a partitioning of threats into clusters (based on machine learning without a teacher) is applied, each of which is mapped to a certain class. Therefore, the work belongs to the S4_T4 group.

The previously described work [106] belongs to the S4_T5 group, and [108] belongs to the S4_T2 group.

## 6. Review Model

A summary of publication reviews (in amount of 85) is presented in four parts (Table 3, Table 4 and Table 5). Columns of the tables have the following designations:

1. Ref. (short for Reference)—link to the publication.
2. Title—publication title.
3. Year—year of publication.
4. Type—type of publication (conference, journal, workshop, lecture notes, report, or preprint).
5. Stage—publication’s affiliation with the corresponding stage of SA.
6. Task—publication’s affiliation with the corresponding ML problem’s solution.
7. Content—main scientific and practical content of the publication, carrying the following meanings (as completed):
  - Theory—full-fledged theory with possible partial realization of a prototype.
  - Experiment—partially realized prototype with full-fledged experiments.
  - Practice—full-fledged prototype or an entire software product.

We examine information from the Table 3, Table 4 and Table 5 in detail in the following order.

**Figure 2:**
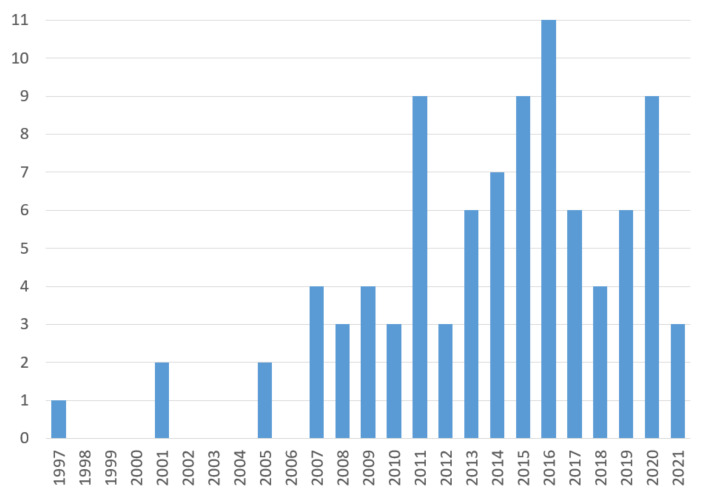
Distribution of publications by year.

First, we analyze publication numbers by year, presented as a histogram in Figure 2.

Thus, first, the minimum year of publication is 1997, and the maximum is 2021, while the average year of publication is 2013. At the same time, after 2007, there is a clear tendency for research in this subject area, although no rapid growth (for example, in recent years) is detected. In the context of the current research task, it can be assumed that some saturation of existing solutions has been achieved in the applying ML for IoTS SA. Therefore, new breakthrough ideas are required for its further development.

**Figure 3:**
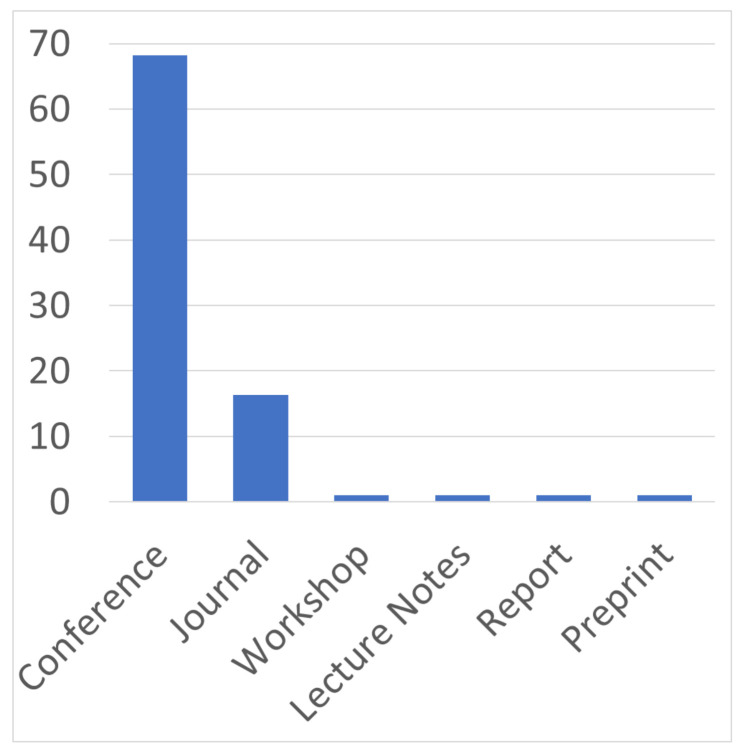
Distribution of publications by type.

Secondly, we analyze the number of publications by type, presented as a bar chart in Figure 3.

The vast majority of the publications belong to conferences, a quarter to journals, and 1 to the other types. The main reason for this distribution is most likely the greater ease of acceptance into a conference proceedings collection than into a journal. Nevertheless, we can reasonably conclude that the scientific community has sufficient awareness of decisions in the field, since information from conferences is more open than from journals. Thus, decisions regarding the use of machine learning for static analysis are of a discussion nature. Consequently, the systematization of all such decisions in this study will accelerate the bringing of the remaining studies to practical implementation. This determines the value of the current survey.

**Figure 4:**
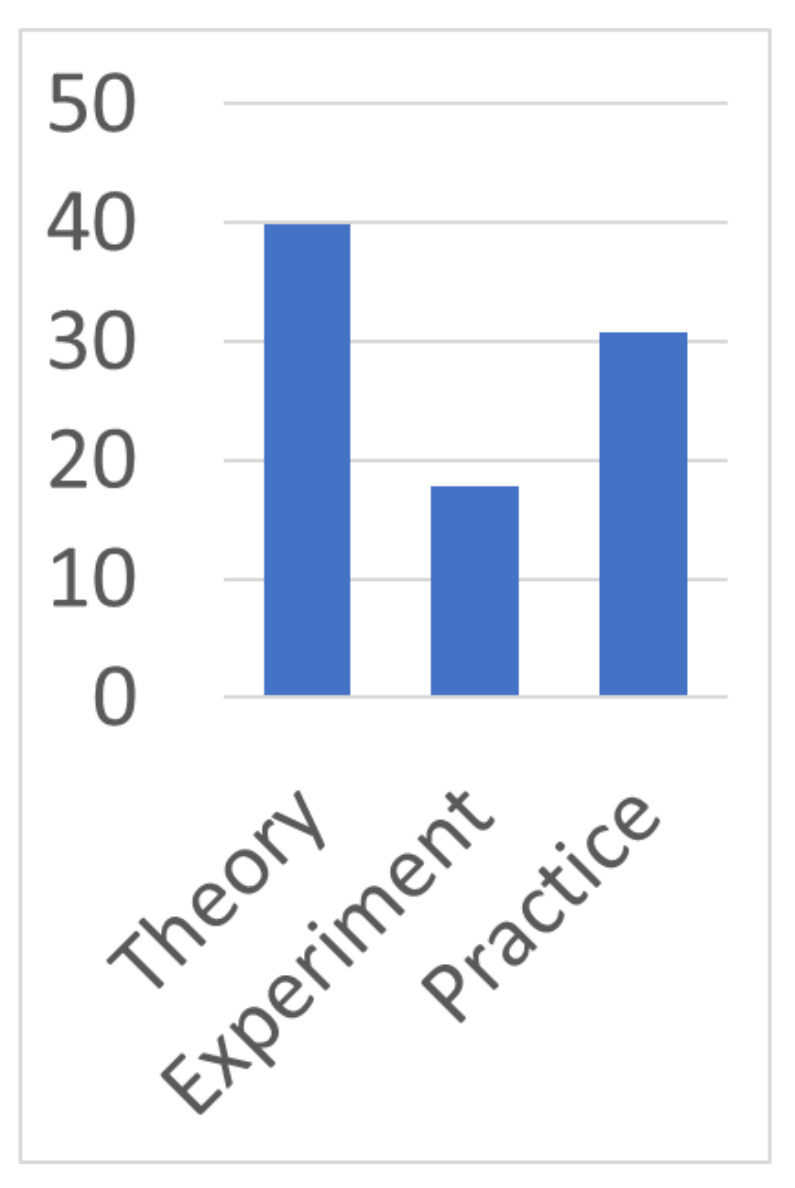
Distribution of publications by content.

Thirdly, we analyze the number of publications according to their scientific and practical content, presented as a bar chart in Figure 4.

We can see that the majority of publications refer more to theoretical research (creation of models and algorithms), although featuring the conduct of certain experiments. Slightly fewer publications are devoted to already finished prototypes, which have passed the minimum necessary testing and are used for experimental evaluations. Substantially fewer publications describe experiments on a prototype with the minimum required functionality. This distribution can be attempted to explain by the fact that the vast majority of the studies, after the theory had been worked out, failed to implement it in practice.

While the part of the research, in which the prototype was nevertheless implemented, successfully brought it to a completed state. Only a small part of the research refused to implement the practical part after conducting experiments. Thus, the following conclusions can be drawn. Bringing the research to its logical conclusion directly depends on the “success” of the choice of the initial idea. The proposed systematization of machine-learning applications for analysis can serve as sources of new, more grounded ideas, supported by already obtained research results.

Based on the above conclusions on results of quantitative analysis by year, type, and content publications, we can draw the following most important conclusion, which also determines the general direction of development in this subject area:

*“While there is sufficient awareness of ML-enabled SA solutions, new breakthrough ideas are needed to evolve IS approaches for IoTS to full-fledged practical products.”*

It is in this direction that the efforts of authors of this review are focused at.

We separately analyze the attribution each of the publications to the SA stage and the ML tasks. In doing so, some of the publications ([56,96,98,99,104,105,106,108,116,117,121,123]) were assigned to several such pairs at once. This would create an appropriate review model, which is presented in matrix form in Table 6. The model describes the works in which ML solutions are applied and which can be applied at each stage of the SA IoTS.

The authors attempted to search and select articles in the following way. The search methodology itself consisted of the following actions: entering a keyword in the *IEEE Explorer*, *ResearchGate*, and *Google Scholar* databases; searching for and studying suitable papers; studying references in papers; searching for new keywords from found papers; etc.

The obtained model has an important methodological value since it almost completely covers the area of study—“SA + ML”. Moreover, it allows us to evaluate the state of elaboration of this area. The search was carried out in two stages—main and advanced.

During the main stage search, the keywords were used that characterize the application of one of the machine-learning tasks for this from the stages of static analysis. Combinations of names of stages and machine-learning tasks (as well as their synonyms or analogs) were used as keywords. For example, to search for the S1_T4 group, the following keywords were selected: “data collection clusterization”, “data gather clusterization”, “data collection cluster algorithm”, etc. Thus, 20 (i.e., 4 Stages *x* 5 Tasks) search passes were made in all databases.

For the keywords of each SA stage and the ML task, a main search was made for scientific publications in each of the scientific databases (IEEE Explorer, ResearchGate, and Google Scholar). Of these, 100 relevant in each of the bases were selected. Then, out of 100 publications, close topics were selected: the current stage of the SA, the solution of the current task of the ML, and the applicability to the IoTS.

In the advanced stage search, instead of the task names (T1–T2), more general keywords were used—machine learning, intelligent, etc. Similarly to the main stage search, the 50 most relevant works were selected for the advanced stage search.

The main criteria for selecting articles for the review was their compliance, both with one of the stages and with one of the tasks. Additional criteria were their application for IoTS analysis. The adaptation of solutions in other areas to this one was taken into account—applying to the IoT.

The total number of found and selected works for each SA stage and ML task is shown in Table 6 and is equal to 91 (including 3 parts of the one investigation—[82,83,84]).

Thus, we can say with some certainty that the resulting distribution of publications in Table 6 reflects the current state and trends. Therefore, a significant amount of research is devoted to the processing of IoTS data in the interest of detecting IS violations by classifying and detecting anomalies (groups S3_T1—22 papers and S3_T2—19 papers), and predicting vulnerabilities using regression (group S3_T3—11 papers), often using generalization (group S3_T5—6 papers).

The attention of scientific papers is focused on the selection of IoTS data using classification (group S1_T1—9 papers). In contrast, for a number of groups (S1_T2, S2_T2, S1_T3, S2_T3, S4_T3, S2_T4, S4_T4, and S2_T5), there were only two studies each. No studies were found on the selection of objects in Stage 1 with the use of dimension reduction (group S1_T5—0 works).

The absence of works for group S1_T5 can be explained by the rarity of the generalization task in IS data collection, nevertheless, such a situation is quite possible; for example, using probabilistic downscaling LSH (Locality-Sensitive Hashing) to find IoTS documents [133] close to a given one, with their merging to apply the other stages of SA.

**Figure 5:**
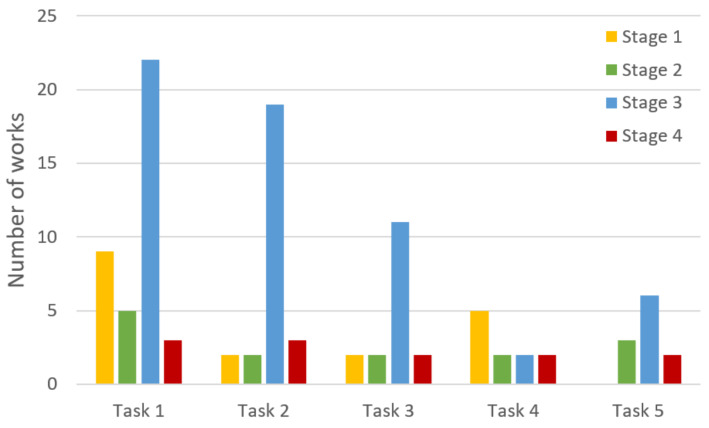
Histogram of research work distribution for static analysis and machine-learning tasks.

Such a distribution of the research work of each SA stage and the ML problem solved in the process is well represented by the histogram in Figure 5.

In the author’s opinion, the uneven distribution of articles in the model is a disadvantage: some subareas appear to be understudied. A number of papers cover several groups at once ([96,98,104,105,106,108,116,117,123]). All this suggests a targeted predisposition of the proposed solutions—capturing a few specific tasks (in each group) out of all of them.

Perhaps it would be more feasible to direct the research towards solving all of the subtasks individually, mapped to each group, i.e., filling each cell of Table 6 completely and evenly. In this way, the possibilities of applying intelligent methods to all groups would have been fully “uncovered”.

The general conclusion from the conducted analysis of scientific publications and their systematization in the form of a table is that for almost every combination of the SA stage and the ML problem there are a number of works substantiating such “symbiosis”. Thus, the second set subtask can be considered solved, which confirms the main hypothesis of the current scientific research from a practical point of view.

A practical suggestion for confirming the hypothesis can be the creation of an intelligent SA framework of IoTS using ML methods. The framework can provide the ability to build complex meta-algorithms for analysis from basic blocks consisting of SA stages. Each of these building blocks can harness the full power of ML. Inputs data for some blocks (i.e., Stage 1) can be outputs from other blocks (i.e., Stage 4). Likewise, a block output (i.e., Stage 4) can serve as an input to other blocks (i.e., Stage 1). Thus, the framework allows building complex SA architectures for large IoTS from minimal ML-based SA blocks and pipes between them.

**Figure 6:**
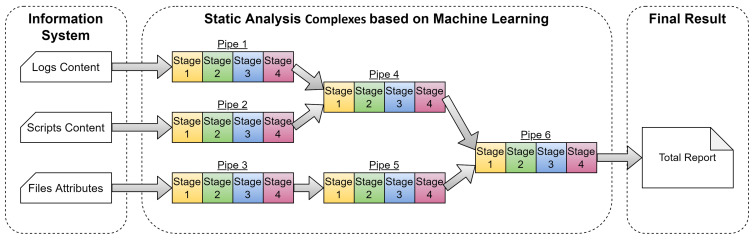
Example of complex static IoTS analysis using machine learning.

An example of a that intellectual framework as a set of interconnected single SA (as part of the described Stage 1–Stage 4) is shown in Figure 6.

The analysis process in Figure 6 is as follows:

1. Content of Logs and Scripts and File Attributes are analyzed by separate Pipes with numbers 1, 2, and 3.
2. Results of the first two Pipes are analyzed by the fourth Pipe and the third Pipe by the fifth Pipe.
3. Results of the fourth Pipe and the fifth Pipe are analyzed by the sixth Pipe, from which a Total Report is created.

Let us justify the possibility of conducting a complex SA based on an intelligent framework (similar to the one shown in Figure 6). Consider again the idea of a four-step process for a particular SA. As input (Stage 1), the SA process takes raw formalized data (usually files). In the main part of the SA (Steps 2 and 3), data is prepared, and problems in information security are searched for. The output of the SA process (Stage 4) generates a representation of the results in the form of formalized data (usually also files). However, the data obtained after Stage 4 may not be sufficient to detect problems on the IoTS.

For example, SA has identified graphs of suspicious interactions between programs, but information security problems themselves have not been detected. In this case, the received data must be subjected to a new SA process (also with four steps). For example, in the graph of interactions between programs, backdoors can be detected using a signature method. Thus, running a SA without any one of the four stages is impractical, and the output from one SA (files after Step 4) can be used as input from another (files from Step 1). Therefore, it makes sense to build an intelligent SA framework as shown in Figure 6.

## 7. Conclusions

In the paper, a theoretical and practical proof of the hypothesis concerning the application of ML to SA was made.

Speaking about the first proof, in a formal form, the execution of each SA Stage and the solution of ML tasks on this stage was considered. Then, the stages and tasks were systematized into a generalized analytical model with a matrix form (see Table 2). The fact of existence and the correctness of the description of this model substantiates the hypothesis from a theoretical point of view. The significance of this scientific result lies in the approbation of the apparatus of formal proofs for solving certain problems by some decisions. The model can serve as the basis for practical implementations of intelligent SA algorithms.

Speaking of the second proof, a review of existing works applicable to the stages of SA of IoTS from the perspective of the tasks solved by ML was made to this end. The results are summarized in a matrix (see Table 6), and a review model was created. The advantage of the model is its necessity and sufficiency—almost all the works refer to its table element. Filling in the cells of the model substantiated the hypothesis from a practical point of view.

The significance of this scientific result lies in the creation of a single consistent base of intelligent solutions (i.e., using ML) in the interests of SA. Thus, if necessary, developers of IoTS analysis systems can make an informed choice of one or another solution for this SA Stage. They can assess the degree of elaboration and technical implementation of the chosen solution path.

The resulting models linking SA and ML allow designing methodological solutions (theoretically and practically justified) in the interest of providing IS of complex IoTS. Naturally, this requires the creation of an appropriate framework to ensure the implementation of all phases using the full variety of ML methods for Big Data and heterogeneous data. An important feature of the framework will be intellectualization supported by the ML methods. As many studies [134,135,136] emphasize, such systems are in great demand in the IoTS IS field.

Despite the availability of reviews regarding ML applications for IoT security (for example, from the point of view of countering attacks [137]), the review and taxonometry proposed in the current article have a number of significant differences. First, the article is devoted specifically to the IoTS analysis but not the attack detection or neutralization. Secondly, the analysis is exactly static (but not dynamic), which allows detecting violations in the system before its immediate launch. Thirdly, a comprehensive consideration of the SA stages and ML tasks allows us to assume not only existing methods of analysis but also hypothetical ones (for example, for a S1_T5). No such reviews have been found in existing scientific papers.

The following directions for further research are proposed.

First, similar to the current review, it is necessary to investigate the possibilities to intellectualize the DA, also breaking it down into phases and introducing solutions from the ML field. As a result, the entire field of IoTS analysis for IS will be fully grasped.

Secondly, based on the overall complexity of SA (as well as DA) large-scale heterogeneous IoTS and their data, a deep and more mathematically correct elaboration of the corresponding mathematical apparatus is required. Although the representation of SA in the form of one of the ML solutions modeled for each stage of SA are given in the article, it is nevertheless more intuitive than objectively correct.

Thirdly, following the concept of an intelligent framework (for the SA of IoTS), it is necessary to create its architecture, synthesis of the basic algorithms, their implementation as a prototype and testing in the “battlefield conditions” (similar to the author’s research [138]). The success of subsequent experiments will prove the functionality of hypothetical framework and allow forming the requirements for its full-fledged development.

## Figures and Tables

**Table 1:** Form and Content transformations in the process of static analysis of the information system.

Transformations	Stage 1. Data Collection	Stage 2. Data Preparation	Stage 3. Data Processing	Stage 4. Result Formation
Form and Content	$\begin{array}{c} <F_{2}|C_{2}>=\\ Stage_{1}\left(<F_{1}|C_{1}>\right) \end{array}$	$\begin{array}{c} <F_{3}|C_{3}{>}^{j}\\ =Stage_{2}^{j}\left(<F_{2}|C_{2}>\right) \end{array}$	$\begin{array}{c} <F_{4}|C_{4}>=\\ Stage_{3}\left(<F_{3}|C_{3}>\right) \end{array}$	$\begin{array}{c} <F_{5}|C_{5}>=\\ Stage_{4}\left(\underset{j=1..M}{⋃}<F_{4}|C_{4}{>}^{j}\right) \end{array}$
Form	$F_{2}=F_{1}$	$F_{3}\neq F_{2}$	$F_{4}\neq F_{3}$	$F_{5}\neq F_{4}$
Content	$|C_{2}|<|C_{1}|$	$\underset{j=1..N}{⋃}C_{3}^{j}\equiv C_{2}$	$C_{4}\neq C_{3}$	$C_{5}=\underset{j=1..M}{⋃}C_{4}^{j}$

**Table 2:** Machine-learning decision matrix for static analysis stages.

Tasks	Stage 1. Data Collection	Stage 2. Data Preparation	Stage 3. Data Processing	Stage 4. Result Formation
Task 1. Classification	$\begin{array}{c} \left\{C\right\}\to \\ \underset{T1}{⋃}{\left\{C\right\}}^{T1},0\times \underset{T2}{⋃}{\left\{C\right\}}^{T2}\\ T1+T2=T \end{array}$	$\begin{array}{c} \left\{F\right\}\to \\ \underset{T1}{⋃}{\left\{F\right\}}^{T1},0\times \underset{T2}{⋃}{\left\{F\right\}}^{T2}\\ T1+T2=T \end{array}$	$\begin{array}{c} \left\{C\right\}\to \\ \underset{T1_{C}}{⋃}{\left\{C\right\}}^{T1_{C}},0\times \underset{T2_{C}}{⋃}{\left\{C\right\}}^{T2_{C}}\\ T1_{C}+T2_{C}=T_{C}\\ \left\{F\right\}\to \\ \underset{T1_{F}}{⋃}{\left\{F\right\}}^{T1_{F}},0\times \underset{T2_{F}}{⋃}{\left\{F\right\}}^{T2_{F}}\\ T1_{F}+T2_{F}=T_{F} \end{array}$	$\begin{array}{c} \left\{F\right\}\to \\ \underset{T1}{⋃}{\left\{F\right\}}^{T1},0\times \underset{T2}{⋃}{\left\{F\right\}}^{T2}\\ T1+T2=T \end{array}$
Task 2. Anomaly detection	$\begin{array}{c} \left\{C\right\}\to {\left\{C\right\}}^{A},0\times {\left\{C\right\}}^{N} \end{array}$	$\begin{array}{c} \left\{F\right\}\to {\left\{F\right\}}^{A},0\times {\left\{F\right\}}^{N} \end{array}$	$\begin{array}{c} \left\{C\right\}\to {\left\{C\right\}}^{A},0\times {\left\{C\right\}}^{N}\\ \left\{F\right\}\to {\left\{F\right\}}^{A},0\times {\left\{F\right\}}^{N} \end{array}$	$\begin{array}{c} c\left\{F\right\}\to {\left\{F\right\}}^{A},0\times {\left\{F\right\}}^{N} \end{array}$
Task 3. Regression	$\begin{array}{c} \left\{C\right\}\to {\left\{D\right\}}^{T1},{0\times \left\{D\right\}}^{T2}\\ T1+T2=T \end{array}$	$\begin{array}{c} \left\{F\right\}\to {\left\{F\right\}}^{T1},{0\times \left\{F\right\}}^{T2} \end{array}$	$\begin{array}{c} \left\{C\right\}\to \\ {\left\{D_{C}\right\}}^{T1_{C}},0\times {\left\{D_{C}\right\}}^{T2_{C}}\\ T1_{C}+T2_{C}=T_{C}\\ \left\{F\right\}\to \\ {\left\{D_{F}\right\}}^{T1_{C}},0\times {\left\{D_{F}\right\}}^{T2_{C}}\\ T1_{F}+T2_{F}=T_{F} \end{array}$	$\begin{array}{c} \left\{F\right\}\to {\left\{F\right\}}^{T1},0\times {\left\{F\right\}}^{T2}\\ T1+T2=T \end{array}$
Task 4. Clustering	$\begin{array}{c} \left\{C\right\}\to \\ \underset{K1}{⋃}{\left\{C\right\}}^{K1},0\times \underset{K2}{⋃}{\left\{C\right\}}^{K2}\\ K1+K2=K \end{array}$	$\begin{array}{c} \left\{F\right\}\to \\ \underset{K1}{⋃}{\left\{F\right\}}^{K1},0\times \underset{K2}{⋃}{\left\{F\right\}}^{K2}\\ K1+K2=K \end{array}$	$\begin{array}{c} \left\{C\right\}\to \\ \underset{K1_{C}}{⋃}{\left\{C\right\}}^{K1_{C}},0\times \underset{K2_{C}}{⋃}{\left\{C\right\}}^{K2_{C}}\\ K1_{C}+K2_{C}=K_{C}\\ \left\{F\right\}\to \\ \underset{K1_{F}}{⋃}{\left\{F\right\}}^{K1_{F}},0\times \underset{K2_{F}}{⋃}{\left\{F\right\}}^{K2_{F}}\\ K1_{F}+K2_{F}=K_{F} \end{array}$	$\begin{array}{c} \left\{F\right\}\to \\ \underset{K1}{⋃}{\left\{F\right\}}^{K1},0\times \underset{K2}{⋃}{\left\{F\right\}}^{K2} \end{array}$
Task 5. Generalization	$\begin{array}{c} \left\{C∼X\right\}\to \\ {\left\{C∼Y\right\}}^{R1},{\left\{C∼Y\right\}}^{R2}\\ R1+R2=R \end{array}$	$\begin{array}{c} \left\{F∼X\right\}\to \\ {\left\{F∼Y\right\}}^{R1},{\left\{F∼Y\right\}}^{R2}\\ R1+R2=R \end{array}$	$\begin{array}{c} \left\{C∼X_{C}\right\}\to \\ {\left\{C∼Y_{C}\right\}}^{R1_{F}},{\left\{C∼Y_{C}\right\}}^{R2_{C}}\\ R1_{C}+R2_{C}=R_{C}\\ \left\{F∼X_{F}\right\}\to \\ {\left\{F∼Y_{F}\right\}}^{R1_{F}},{\left\{F∼Y_{F}\right\}}^{R2_{F}}\\ R1_{F}+R2_{F}=R_{F} \end{array}$	$\begin{array}{c} \left\{F∼X\right\}\to \\ {\left\{F∼Y\right\}}^{R1},{\left\{F∼Y\right\}}^{R2}\\ R1+R2=R \end{array}$

Note. In the table, the expression with multiplication by zero “0 *x*” means that in the process of applying the solution from the ML field data were obtained, which are not used to this end of SA at this stage. Thus, some classes {*C*}, regression numbers {*D*}, clusters {*O*}^*K*^ and reduced feature dimensions {O ∼ Z} are divided into two sets (with indices T1 and T2 and a common T, with indices K1 and K2 and a common K, and with indices R1 and R2 and a common R), the second of which is not used in the interest of SA.

**Table 3:** Publication summary (Part 1).

Ref.	Title	Year	Type	Stage	Task	Content
[58]	Toward Large-scale Vulnerability Discovery Using Machine Learning	2016	Conference	3	1	Practice
				3	3	
[110]	Detection of malicious code by applying machine-learning classifiers on static features: A state-of-the-art survey	2009	Journal	3	1	Experiment
[111]	Malicious Code Detection Using Active Learning	2008	Conference	3	1	Theory
[100]	Type Learning for Binaries and Its Applications	2019	Conference	2	1	Experiment
[81]	Method for classification of files based on machine-learning technology	2020	Journal	1	1	Practice
[82,83,84]	Identification of Processor’s Architecture of Executable Code Based on Machine Learning	2020	Journal	1	1	Practice
[101]	Machine Learning-Assisted Binary Code Analysis	2007	Workshop	2	1	Theory
[122]	o-glasses: Visualizing x86 Code from Binary Using a 1d-CNN	2020	Conference	4	1	Theory
[112]	Cyber Vulnerability Intelligence for Internet of Things Binary	2020	Conference	3	1	Experiment
[113]	A machine-learning approach to anomaly-based detection on Android platforms	2015	Journal	3	2	Practice
[114]	Android malware detection using the dendritic cell algorithm	2014	Conference	3	2	Experiment
[86]	Similarity detection among data files—a machine-learning approach	1997	Conference	1	4	Theory
[88]	Document Clustering for Forensic Computing: An Approach for Improving Computer Inspection	2011	Conference	1	4	Experiment
[87]	Document Clustering—A Feasible Demonstration with K-means Algorithm	2019	Conference	1	4	Theory
[102]	Evolution in Software Architecture Recovery Techniques—A Survey	2017	Conference	2	4	Theory
[103]	A Hierarchical Clustering-Based Approach for Software Restructuring at the Package Level	2017	Conference	2	4	Practice
[123]	A Novel Solutions for Malicious Code Detection and Family Clustering Based on Machine Learning	2019	Conference	4	1	Theory
				4	4	
				4	5	
[115]	Android malware detection using 3-level ensemble	2016	Conference	3	5	Experiment
[104]	Reverse engineering smart card malware using side channel analysis with machine-learning techniques	2016	Conference	2	5	Theory
				3	5	
[116]	Feature selection and machine-learning classification for malware detection	2015	Journal	3	1	Theory
				3	5	
[117]	Android malware detection based on permissions	2014	Conference	3	1	Practice
				3	5	
[106]	Android ransomware detection using reduced opcode sequence and image similarity	2017	Conference	2	5	Experiment
				4	5	
[85]	File Block Classification by Support Vector Machine	2011	Conference	1	1	Theory
[118]	Preventing File-Less Attacks with Machine Learning Techniques	2019	Conference	3	2	Theory
[89]	Document Image Classification and Labeling using Multiple Instance Learning	2011	Conference	1	1	Experiment
[90]	Multi-scale Structural Saliency for Signature Detection	2007	Conference	1	1	Practice
[91]	Multi-instance clustering with applications to multi-instance prediction	2009	Journal	1	4	Experiment
[92]	Detection of packed executables using support vector machines	2011	Conference	1	1	Practice
[93]	Detecting Packed Executable File: Supervised or Anomaly Detection Method?	2016	Conference	1	2	Experiment
[94]	An anomaly detection system proposal to ensure information security for file integrations	2018	Conference	1	2	Theory
[124]	Visualizing Big Data Outliers through Distributed Aggregation	2018	Conference	4	2	Theory

**Table 4:** Publication summary (Part 2).

Ref.	Title	Year	Type	Stage	Task	Content
[128]	Relational Synthesis of Text and Numeric Data for Anomaly Detection on Computing System Logs	2016	Conference	4	2	Practice
[95]	Predicting File Lifetimes with Machine Learning	2019	Lecture Notes	1	3	Theory
[107]	A Machine-Learning Approach to Automatic Detection of Delimiters in Tabular Data Files	2016	Conference	2	3	Theory
[96]	Multiple linear regression for universal steganalysis of images	2018	Conference	1	3	Experiment
				2	3	
[108]	Log File Anomaly Detection	2016	Report	2	2	Theory
				4	2	
[109]	Experimentations with OpenStack System Logs and Support Vector Machine for an Anomaly Detection Model in a Private Cloud Infrastructure	2020	Conference	2	2	Experiment
[130]	Forecasting Zero-Day Vulnerabilities	2016	Conference	4	3	Practice
[131]	The Effects of Depth of Field on Subjective Evaluation of Aesthetic Appeal and Image Quality of Photographs	2020	Journal	4	3	Experiment
[97]	Text Document Classification with PCA and One-Class SVM	2017	Conference	1	1	Theory
[119]	Machine Learning With Feature Selection Using Principal Component Analysis for Malware Detection—A Case Study	2019	Journal	3	1	Theory
[105]	Power-based Side-Channel Instruction-level Disassembler	2018	Conference	2	5	Practice
				3	5	
[17]	Data mining methods for detection of new malicious executables	2001	Conference	3	1	Practice
[18]	Integrated static and dynamic analysis for malware detection	2015	Journal	3	1	Experiment
[19]	Classification of malware families based on N-grams sequential pattern features	2013	Conference	3	1	Experiment
[20]	Malware detection using machine learning	2009	Conference	3	1	Practice
[21]	Byteweight: Learning to recognize functions in binary code	2014	Conference	2	1	Practice
[22]	Recognizing functions in binaries with neural networks	2015	Conference	2	1	Theory
[23]	Automatically learning semantic features for defect prediction	2016	Conference	3	2	Theory
[24]	Emergent, crowd-scale programming practice in the IDE	2014	Conference	3	2	Practice
[25]	Using web corpus statistics for program analysis	2014	Conference	3	2	Practice
[26]	Bugram: bug detection with n-gram language models	2016	Conference	3	2	Practice
[27]	Finding Likely Errors with Bayesian Specifications	2017	Preprint	3	2	Practice
[28]	Learning to Represent Programs with Graphs	2018	Conference	3	2	Theory
[29]	Deep Learning to Find Bugs	2017	Journal	3	2	Practice
[30]	Strengthening the empirical analysis of the relationship between linus’ law and software security	2010	Conference	3	3	Theory
[31]	An empirical study of the evolution of PHP web application security	2011	Conference	3	3	Theory
[32]	Can traditional fault prediction models be used for vulnerability prediction?	2013	Journal	3	3	Theory
[33]	An initial study on the use of execution complexity metrics as indicators of software vulnerabilities	2011	Conference	3	3	Theory
[34]	Evaluating complexity, code churn, and developer activity metrics as indicators of software vulnerabilities	2011	Journal	3	3	Theory
[35]	Using complexity metrics to improve software security	2013	Journal	3	3	Theory
[36]	Predicting vulnerable components: Software metrics vs text mining	2014	Conference	3	3	Theory

**Table 5:** Publication summary (Part 3).

Ref.	Title	Year	Type	Stage	Task	Content
[37]	Challenges with applying vulnerability prediction models	2015	Conference	3	3	Theory
[38]	To fear or not to fear that is the question: Code characteristics of a vulnerable function with an existing exploit	2016	Conference	3	3	Theory
[39]	Searching for a needle in a haystack: Predicting security vulnerabilities for windows vista	2010	Conference	3	3	Theory
[40]	Bugs as deviant behavior: A general approach to inferring errors in systems code	2001	Conference	3	2	Practice
[41]	DynaMine: Finding common error patterns by mining software revision histories	2005	Conference	3	2	Practice
[42]	PR-miner: Automatically extracting implicit programming rules and detecting violations in large software code	2005	Conference	3	2	Practice
[43]	Detecting object usage anomalies	2007	Conference	3	2	Practice
[44]	Mining API patterns as partial orders from source code: From usage scenarios to specifications	2007	Conference	3	2	Practice
[45]	Alattin: Mining alternative patterns for detecting neglected conditions	2009	Conference	3	2	Theory
[46]	Learning from 6000 projects: Lightweight cross-project anomaly detection	2010	Conference	3	2	Theory
[47]	Discovering neglected conditions in software by mining dependence graphs	2008	Journal	3	2	Theory
[48]	Chucky: Exposing missing checks in source code for vulnerability discovery	2013	Conference	3	2	Theory
[49]	Vulnerability extrapolation: Assisted discovery of vulnerabilities using machine learning	2011	Conference	3	1	Experiment
[50]	Generalized vulnerability extrapolation using abstract syntax trees	2012	Conference	3	1	Theory
[51]	Predicting common web application vulnerabilities from input validation and sanitization code patterns	2012	Conference	3	1	Experiment
[52]	Predicting SQL injection and cross site scripting vulnerabilities through mining input sanitization patterns	2013	Journal	3	1	Practice
				3	4	
[53]	Mining SQL injection and cross site scripting vulnerabilities using hybrid program analysis	2013	Conference	3	1	Experiment
[54]	Web application vulnerability prediction using hybrid program analysis and machine learning	2015	Journal	3	1	Theory
[55]	Predicting vulnerable software components via text mining	2014	Journal	3	1	Theory
[56]	Automatic inference of search patterns for taintstyle vulnerabilities	2015	Conference	3	1	Experiment
				3	4	
[57]	Predicting vulnerable software components through N-gram analysis and statistical feature selection	2015	Conference	3	1	Theory
[120]	Classification and Analysis of Android Malware Images Using Feature Fusion Technique	2021	Conference	3	1	Practice
[121]	SHELLCORE: Automating Malicious IoT Software Detection Using Shell Commands Representation	2021	Conference	3	1	Practice
				3	5	
[98]	Machine Learning Tensor Flow Based Platform for Recognition of Hand Written Text	2021	Conference	1	1	Practice
				2	1	
[99]	A Machine Learning-Based Framework for Mobile Forensics	2020	Conference	1	1	Practice
				1	4	
[129]	Automation of Vulnerability Classification from its Description using Machine Learning	2020	Conference	4	1	Practice
[132]	Threats Classification Method for the Transport Infrastructure of a Smart City	2020	Conference	4	4	Theory

**Table 6:** Overview model of scientific works on the implementation of the static analysis stages using machine learning.

Task Name	Stage 1. Data Collection	Stage 2. Data Preparation	Stage 3. Data Processing	Stage 4. Result Formation
Task 1. Classification	[81,82,83,84,85,89,90,92,97,98,99]	[21,22,98,100,101]	[17,18,19,20,49,50,51,52,53,54,55,56,57,58,110,111,112,116,117,119,120,121]	[122,123,129]
Task 2. Anomaly detection	[93,94]	[108,109]	[23,24,25,26,27,28,29,40,41,42,43,44,45,46,47,48,113,114,118]	[108,124,128]
Task 3. Regression	[95,96]	[96,107]	[30,31,32,33,34,35,36,37,38,39,58]	[130,131]
Task 4. Clustering	[86,87,88,91,99]	[102,103]	[52,56]	[123,132]
Task 5. Generalization		[104,105,106]	[104,105,115,116,117,121]	[106,123]

## Data Availability

Data sharing is not applicable to this article.
